# The significance of anatomical variation of the inferior mesenteric artery and its branches for laparoscopic radical resection of colorectal cancer: a review

**DOI:** 10.1186/s12957-022-02744-6

**Published:** 2022-09-10

**Authors:** Shun Zeng, Wenhao Wu, Xianbin Zhang, Tong Qiu, Peng Gong

**Affiliations:** 1 Department of General Surgery & Institute of Precision Diagnosis and Treatment of Gastrointestinal Tumors, Shenzhen University General Hospital & Shenzhen University Clinical Medical Academy, Xueyuan Road 1098, Shenzhen, 518055 China; 2 Carson International Cancer Center & Guangdong Provincial Key Laboratory of Regional Immunity and Diseases, Shenzhen University Health Science Center, Xueyuan Road 1066, Shenzhen, 518060 China; 3 Shenzhen University Clinical Medical Academy, Xueyuan Road 1066, Shenzhen, 518060 China

**Keywords:** Laparoscopic resection of colorectal cancer, Vascular anatomy, Colorectal cancer, Inferior mesenteric artery, Left colonic artery, Classification, Riolan artery arch

## Abstract

Currently, high or low ligation of the inferior mesenteric artery (IMA) is a controversial issue in laparoscopic radical surgery for colorectal cancer. High or low ligation of the IMA has both advantages and disadvantages, and the level of ligation during the left colon and/or rectum resection has been a dilemma for surgeons. One important factor influencing the surgeon’s decision to ligate the IMA in a high or low position is the anatomical type of the IMA and its branches. Some studies confirm that the anatomy of the IMA and its branches is critical to the anastomotic blood supply and, therefore, influences the choice of surgical approach (level of ligation of the IMA). However, many vascular variations in the anatomy of the IMA and its branches exist. Herein, we have summarized the anatomical types of the IMA and its branches, finding that the classification proposed by Yada et al. in 1997 is presently accepted by most scholars. Based on Yada’s classification, we further summarized the characteristics of the IMA’s various anatomical types as a guide for high or low ligation in radical colorectal cancer surgery.

## Background

The left colic artery (LCA) is the uppermost branch of the inferior mesenteric artery, starting 2–3 cm from the root of the inferior mesenteric artery and traveling leftward to the deep surface of the peritoneum wall. It divides into ascending and descending branches, which nourish the left flexure of the colon and the descending colon, and anastomoses with branches of the middle colic artery and sigmoid artery, respectively. The inferior mesenteric artery (IMA) originates from the anterior surface of the aorta behind the lower border of the duodenum and is located 3–4 cm above the level of the aortic bifurcation L2–L3. The LCA is the first branch of IMA. The IMA and its vessels are among the most important anatomical landmarks in colorectal surgery. Controversy exists on the use of high or low ligation of the IMA in laparoscopic radical surgery for colorectal cancer. One of the important factors influencing the surgeon’s decision to ligate the IMA in a high or low position is the anatomical type of the inferior mesenteric artery and its branches. Some studies [1–11] corroborate that the anatomy of the IMA and its branches is critical to the anastomotic blood supply, therefore influencing the choice of surgical approach (level of IMA ligation). However, owing to the narrow laparoscopic view and lack of palpation, vascular bifurcation variants can easily be misdiagnosed as injuries, causing serious complications such as hemorrhage and intestinal ischemia. Pre-operative knowledge of arterial branching or variants, including the characteristics of each type, is useful for surgeons in developing pre-operative strategies for safe, rapid vascular ligation and lymph node dissection. This review summarizes the anatomical types of IMA and its branches and discusses the significance of the Riolan arterial arch, with the aim of providing guidance for precise ligation of the vessels during laparoscopic radical colorectal cancer surgery.

## Material and methods

In this study, we searched the National Institute of Health PubMed database using a combination of subject and free words, including “Cancer,” “Tumor,” “Colon,” “Rectum,” “Colorectal,” “Ligation,” “Anatomical variation,” “Inferior mesenteric artery,” “IMA,” “Left colonic artery,” “LCA,” and “Riolan” as keywords. A total of 303 references were identified during an initial search of the PubMed database, and 11 additional references were identified by a manual search. After excluding duplicate citations and carefully reviewing abstracts, 53 papers were selected for a full-text review. In total, 48 studies were included in the final review.

### Advantages and disadvantages of ligating the IMA in high and low positions

In recent years, laparoscopic resection of colorectal cancer has gained widespread clinical acceptance. With continuous improvements in laparoscopic techniques, consensus was reached on many aspects of surgical treatment, including total mesorectal excision, lymph node dissection of the IMA root, and preservation of pelvic autonomic nerves. The 2019 Japanese Society for Colorectal Cancer Research (JSCCR) guidelines recommend that lymph node dissection in progressive rectal cancer should include the root of the inferior mesenteric vessels; however, these guidelines do not specify the site of IMA ligation [12]. Likewise, the National Comprehensive Cancer Network (USA) guidelines do not indicate whether the LCA should be preserved [13]. Therefore, the ligation level (high or low) of the IMA during the left colon and/or rectal resection is unelucidated. Low ligation is the separation and ligation of the branches of the left colonic artery, whereas high ligation is the separation and ligation of the aorta at its origin. Therefore, by definition, low ligation will preserve the left colonic artery, whereas high ligation does not. In laparoscopic radical colorectal cancer surgery, ligation of the IMA at high and low positions has both advantages and disadvantages. Proponents of high ligation believe that it can better clear root lymph nodes, reduce anastomotic tension, provide accurate tumor staging, and preserve the nerves. This enables patients to achieve longer overall survival, minimizing the risk of tumor cell spillage and local recurrence [14–20], In comparison, low ligation has insufficient lymph node clearance and thus increases the probability of metastatic recurrence. Proponents of low ligation believe that high ligation will reduce the proximal blood supply to the anastomosis because of root ligation, increasing the risk of anastomotic leakage, and damage to autonomic nerve function [21–32]. Low ligation after IMA can increase the proximal blood supply to the anastomosis and protect autonomic nerve function because the left colonic artery is preserved. It is also relatively simple to perform. In this case, the anatomy of the IMA and its branches is very important for radical colorectal cancer surgery, and anatomical structures should be operated on differently. Accurate recognition and intra-operative assessment of the anatomy and possible changes in IMA and LCA are crucial when performing radical resection for colorectal tumors.

### Anatomical types of IMA and its branches and Riolan arterial arch

**Fig. 1:**
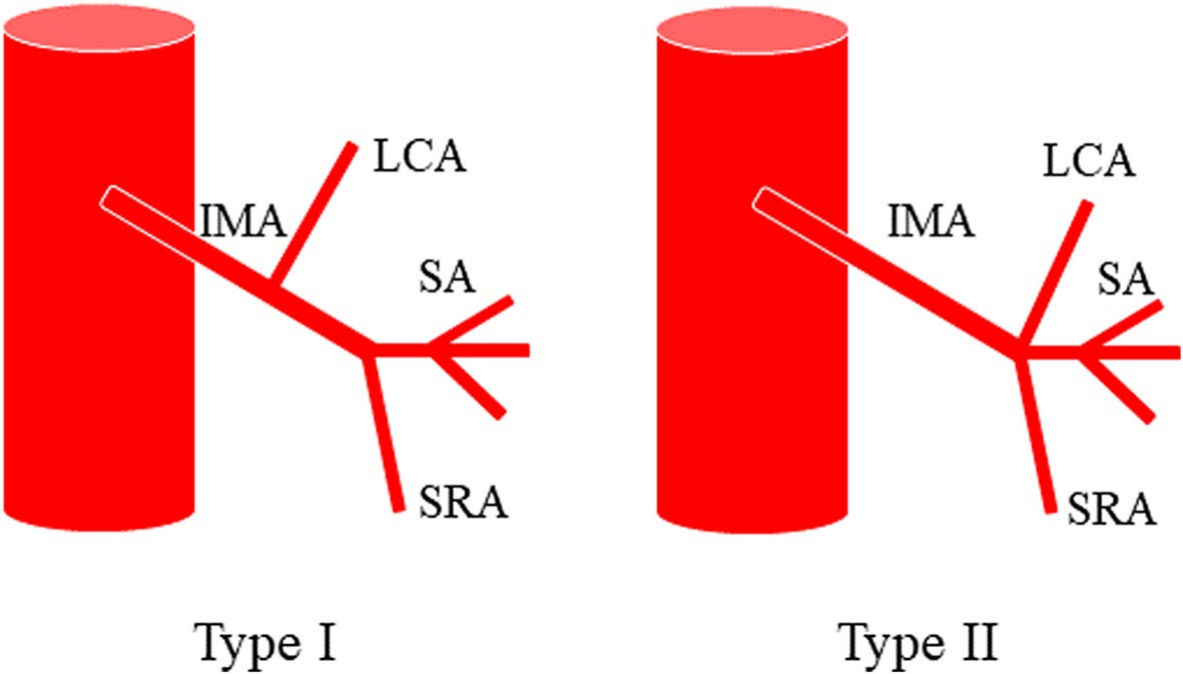
Laterjet’s classification of the branching type of the inferior mesenteric artery: type I (spread) and type II (fan)

**Fig. 2:**
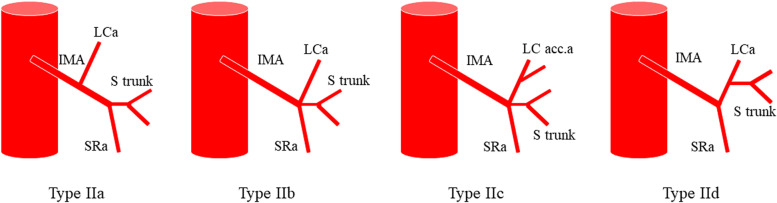
Predescu’s classification of the branching pattern of the IMA (IMA, inferior mesenteric artery; LCa, left colic artery; S trunk, sigmoid artery trunk; LC acc.a, middle left colonic artery MLCA or the inferior left colonic artery ILCA; SRa, superior rectal artery)

Numerous anatomical variants exist of the IMA and its branches, and there is no unified anatomical typing standard. In 1949, Latarjet [33] described two types of anatomical variants of the IMA (Fig. 1): type I: independent origin, the LCA and sigmoid artery (SA) have separate origins and type II: fan-shaped origin, the LCA and SA co-trunk share a common origin. Later, Predesc [34] added to Latarjet’s classification (Fig. 2) and described the following types: type I: identical to Latarjet type I and types IIa, IIb, IIc, and IId: subdivisions of Latarjet type II, defined as follows: IIa: IMA divides into LCA, SA, and superior rectal artery (SRA); IIb: LCA, SA, SRA emanate from the same origin; IIc, on the basis of IIb, LCA divides into the middle left colonic artery (MLCA) or inferior left colonic artery (ILCA); and IId, LCA, and SA co-trunk.

**Fig. 3:**
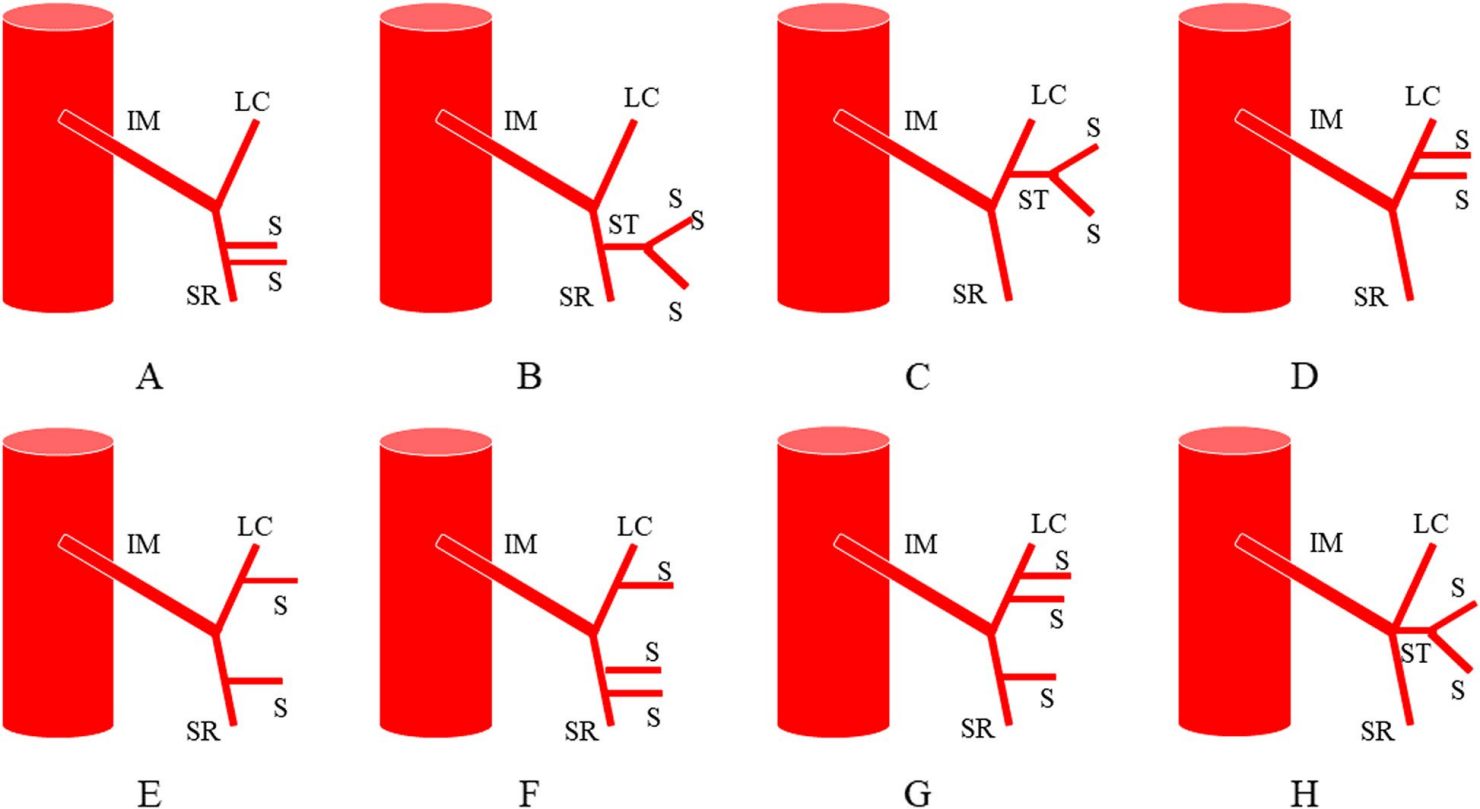
Zebrowski classification: C, common artery; IM, inferior mesenteric artery; LC, left colonic artery; RST, rectosigmoid trunk; SR, superior rectal artery; ST, sigmoid trunk

**Fig. 4:**
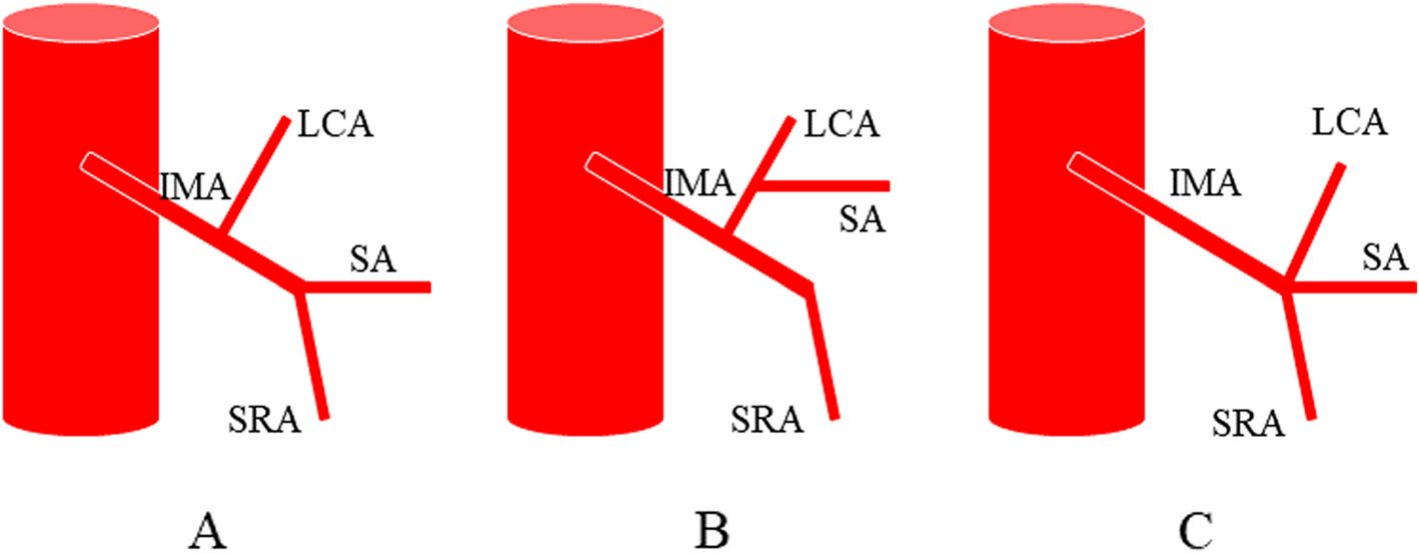
Wang’s classification of inferior mesenteric artery (IMA) branching types into A-C (AA, abdominal aorta; LCA, left colonic artery; SA, sigmoid artery; SRA, superior rectal artery)

**Fig. 5:**
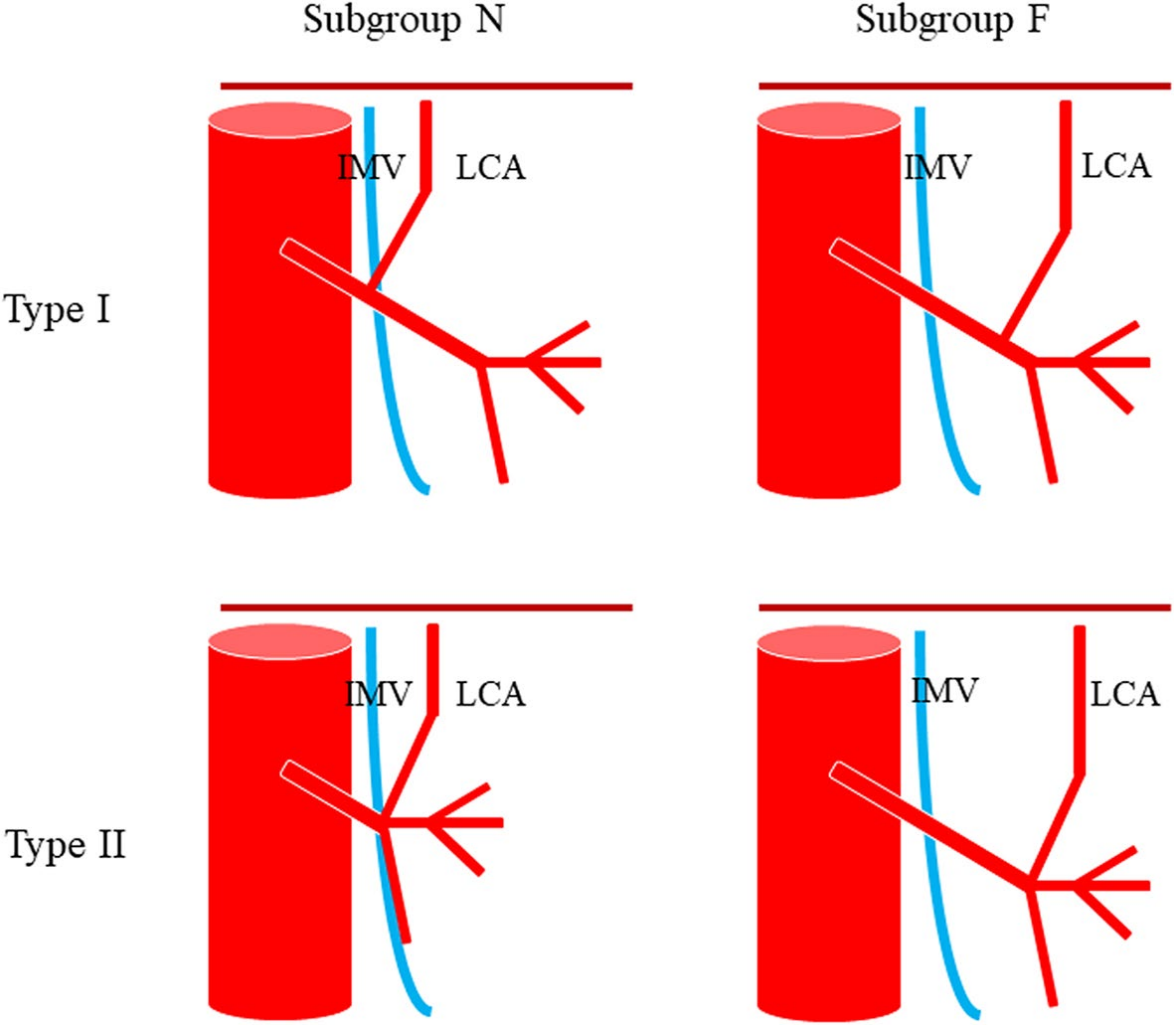
Classification of Patroni. Type I and type II represent diffuse or fan-shaped IMA branching patterns, respectively (Laterjet typing). Subgroup N represents IMV-LCA distances greater or less than 20 mm at the inferior margin of the pancreas, respectively; subgroup F represents IMV-LCA distances greater than 20 mm at the inferior margin of the pancreas, respectively

**Fig. 6:**
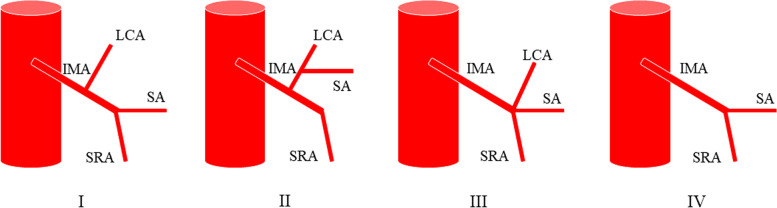
Yada classification (I: LCA emanates from IMA independently; II: LCA and SA co-trunk; III: LCA, SA and SRA emanate from the same point; IV: LCA is absent)

In 1971, Zebroski [35] (Fig. 3) classified IMA anatomic variants into eight types: (A) IMA first emits LCA, two SAs have a common trunk with SRA, respectively; (B) IMA first emits LCA, two SAs share a common trunk (ST), ST co-trunks with SRA; (C) IMA first emits LCA, two SAs share a common trunk ST, ST co-trunks with LCA; (D) IMA first emits LCA and two SAs have a common trunk with LCA, respectively; (E) IMA first emits LCA, two SAs have a common trunk with LCA and SRA, respectively; (F) IMA first emits LCA, three SAs, one of which has a common trunk with LCA and the other two have a common trunk with SRA, respectively; (G) IMA first emits LCA, three SAs, one of which has a common trunk with SRA and the other two have a common trunk with LCA, respectively; and (H) LCA, SA, and SRA emanate from a common starting point. In recent years, Wang [7] (Fig. 4) classified anatomical types into three types: type A: LCA arises independently of the IMA; type B: LCA and SA branches from a common IMA trunk; and type C: LCA, SA, and SRA branches from the IMA at the same point. Miyamoto [2] classified IMA anatomical typology into three types: type A: LCA and SA bifurcate from the same point of the IMA; type B: the common trunk of the LCA and SA separates from the IMA; and type C: the LCA and SA separate from the IMA. Patroni [5] (Fig. 5) divided Latarjet typing into groups *N* and *F* (*N*: < 20 mm, *F*: ≥ 20 mm) based on the distance between the LCA and IMV at the inferior margin of the pancreas. Many current typing methods have a more limited role in guiding high/low ligation of the IMA during laparoscopic radical surgery for colorectal cancer, creating difficulties in preserving or further identifying and preserving the LCA. Latarjet [33] was the first to propose anatomical variants and typing of IMA and LCA, but its division into two types does not provide a good overview of all types of anatomical variants of IMA and LCA. Predescu [34] further elaborated the typing based on Latarjet’s classification and introduced the concept of the left middle colic artery (MLCA), which nicely complemented Latarjet’s classification. Zebroski [35] classified the anatomical variants of the IMA into eight types, but its overly detailed typing was not conducive to the surgeon’s identification of the target vessels pre-operatively and intra-operatively. Wang’s typing [7] aptly summarized the characteristics of anatomical variants of the IMA and LCA but lacked discussion regarding the absence of LCA. Patroni’s typing method [5] introduced the concept of the submesenteric vein but did not discuss the anatomical variants of the IMA and LCA in detail. These typing methods are not conducive to the surgeon’s precise understanding of their anatomical structures and judgment of the intra-operative ligature location of the vessels, making it difficult to identify and preserve the LCA during laparoscopic radical surgery for colorectal cancer. Therefore, they are not conducive to reducing the incidence of post-operative complications. Yada, a Japanese researcher, was the first to classify IMA in 1997 based on the relationship between LCA, SA, and the root initiation point of SRA [8] (Fig. 6). The four types of typing were accepted by most researchers, and most clinical studies since then have been based on this as it better presents the relationship between IMA, LCA, SA, and SRA. It is helpful for surgeons to precisely recognize the anatomy pre-surgery, thereby they can choose the correct operation and decide whether to preserve the left colon. I: LCA emanates from IMA independently; II: LCA and SA co-trunk; III: LCA, SA, and SRA emanate from the same point; and IV: LCA is absent.

Under the Yada classification, the percentage occurrence of each type in the nine papers was counted, as shown in Table 1. According to the statistical results, the probability of occurrence of type I ranged from 32.1 to 59.4%, type II from 10.3 to 53.6%, type III from 8.5 to 44.7%, and type IV from 0 to 2.8%.

**Table 1:** Statistics on the number of cases and proportion of various subtypes present in the nine studies

	I	II	III	IV	Total
Bertrand et al. [4]	41(44.1%)	21(47.5%)	17(18.3%)	7 (7.5%)	93
Ke et al. [1]	89(47.3%)	39(20.7%)	51(27.13%)	9 (4.8%)	188
Kobayashi et al. [6]	27 (32.1%)	45 (53.6%)	10 (11.9%)	2 (2.3%)	84
Miyamoto et al. [2]	20 (43.5%)	21 (45.7%)	5 (10.8%)	0 (0)	46
Murono et al. [36]	193 (41.2%)	42 (9.0%)	209 (44.7%)	24 (5.1%)	468
Wang et al. [7]	51 (46.3%)	26 (23.6%)	33 (30.0%)	0 (0)	110
Zhang et al. [3]	63 (59.4%)	31 (29.2%)	9 (8.5%)	3 (2.8%)	106
Huang et al. [10]	67 (57.8%)	12 (10.3%)	36 (31.0%)	1 (0.9%)	116
Zhou et al. [11]	58 (55.7%)	12 (11.5%)	32 (30.7%)	2 (1.9%)	104

### The significance of IMA branch classifications

Type I: Luo et al. [37] concluded that in patients with colorectal cancer with type I IMA, low ligation can be performed when the LCA origin is revealed, preserving the blood supply and reducing unnecessary operative time. According to Patroni [5], a single LCA origin was observed in 71% of cases (types I, II), and since the LCA is more easily preserved at the origin, this is considered a good outcome of low ligation (preserved LCA). This study concluded that in more than two-thirds of cases, the preservation of the LCA at its origin is highly feasible because of its different starting points. In addition, several studies [1, 4, 6, 7, 36] have found that the average distance of LCA initiation from IMA initiation was closer in type I patients than in other IMA types, which could impact the extent of intraoperative lymph node dissection, a crucial point to consider during the procedure.

Type II: Huang et al. [10] concluded that the LCA originates far from the root of the IMA and co-stems with the SA, which is easier to locate during laparoscopic surgery and can be considered for radical colorectal cancer surgery with preservation of the LCA. Luo et al. [37] concluded that for type II IMA, LCA preservation will likely lead to difficulty in proximal bowel pull-down, increase the anastomotic tension, and increase the possibility of anastomotic leakage.

Type III: According to Huang et al. [10], type III is easier to detect and separate because LCA, SA, and SRA are co-interstitial. In type III, the LCA divides from the SA and SRA at the same point low in the IMA and moves towards the descending colon. The blood supply to the splenic flexure of the colon and descending colon is supplied only by the marginal arch between the left branch of the middle colonic artery and the LCA. Moreover, the descending colon has a longer segment of the intestinal canal and lacks a direct blood supply from the tertiary arteries. Therefore, patients with colorectal cancer with type III vascular dissection should undergo radical colorectal cancer surgery with preservation of the LCA because high ligation of the IMA during radical resection will block LCA blood flow and may lead to inadequate blood supply to the descending colon and distal anastomosis, thus increasing the risk of anastomotic leakage. However, according to Luo et al. [37], for patients with type III IMA, it is imperative to reveal the SRA, SA, and LCA common trunk, including dissociating the sigmoid and superior colorectal arteries. This dissociation should be performed with extra care because incorrect surgery can easily lead to LCA bleeding and necessitate abandoning low ligation, reducing the anastomotic blood supply.

The Riolan arterial arch, which is the anastomotic branch between the ascending branch of the LCA and left branch of the middle colonic artery, has been described in several studies. In a study by Huang et al. [10], 60.3% (70/116) of the Riolan arterial arches were absent, with 49.3% (33/67), 83.3% (10/12), 72.2% (26/36), and 100% (1/1) of the IMA types absent, respectively. However, these differences were not statistically significant (*P* = 0.125). This study concluded that type III and Riolan arterial arch defects are independent risk factors for the development of anastomotic leakage. In this study, no anastomotic leakage occurred after high ligation in type III patients with a Riolan arch, whereas all patients with a type III anastomotic leakage had a combined Riolan arch defect. In patients with an absent Riolan arch, left hemicolectomy is dependent on the IMA for blood supply, and the high-ligation technique can possibly cause ischemic changes in the anastomosis. Therefore, a low-ligation technique that preserves the LCA in order to maintain blood supply is recommended. Low ligation with highly selective lymph node dissection may be considered for patients with type III and Riolan artery arch agenesis. Wang et al. [7] concluded that Riolan artery arch agenesis is an independent risk factor for anastomotic leakage after laparoscopic radical colorectal cancer surgery. Since left hemicolectomy relies on the IMA for blood supply in cases of Riolan artery arch deficiency, the use of high ligation leads to ischemic changes in the anastomosis, thereby increasing the risk of postoperative anastomotic leakage.

## Discussion

In the laparoscopic resection of colorectal cancer, high/low ligation of the IMA remains controversial. Tumor staging is the basis of colorectal cancer treatment and determines the choice of treatment strategy as well as the ability to preserve LCA. Thorough lymph node dissection is the key to radical resection and accurate tumor staging of colorectal cancer. A study [38] showed that patients with stage T1 rectal cancer had no metastasis in group 253 lymph nodes, and the metastasis rate was 0.95% (1/105) in stage T2, 5.22% (6/115) in stage T3, and 6.12% (12/196) in stage T4, suggesting that metastasis in group 253 lymph nodes might theoretically exist in all rectal cancers above stage T2. Therefore, whether the group 253 lymph nodes can be completely cleared is the key to the preservation of the LCA procedure and the main point of controversy regarding the preservation of the LCA. High ligation of the IMA provides effective, complete, and intact clearance of group 253 lymph nodes, but it is not the only method for complete clearance of this group of lymph nodes. Studies have shown no statistically significant difference in the clearance of 253 lymph nodes in patients with and without retained LCA [21, 23, 25, 26, 28–32]. Both retrospective and prospective studies have shown that high and low ligation in rectal cancer surgery has comparable effects on overall and recurrence-free survival, with no statistically significant differences in survival even in patients with lymph node metastases [11, 39, 40]. The oncological safety of the preserved LCA procedure was confirmed. However, it is worth noting that in patients with excessive metastases and fusion of group 253 lymph nodes into clusters, dissection to reveal the LCA and preservation of the LCA can pose technical challenges and increase the probability of intra-operative bleeding. Therefore, for patients found to be in this category on intra-operative exploration, preservation of the LCA is not recommended, and high IMA ligation is feasible to reduce the surgical difficulty and ensure oncological safety [41]. According to a recent expert consensus published in 2021 [41], the decision to preserve the LCA depends on various factors, including rectal and partial colon cancer, older adults, combined metabolic diseases, neoadjuvant therapy, risk of multiple primary colorectal cancers, and persistent descending mesocolon (PDM). There are numerous reasons for this. Advanced age and diabetes mellitus are recognized as high-risk factors for anastomotic leakage [42, 43]. In resected colorectal cancer specimens after radiotherapy, the peri-cancerous tissue mucosa, submucosa, and surrounding adipose tissue show varying degrees of inflammatory changes and fibrosis and the colorectal mucosa and mesocolon tissue show microvascular damage and increased fragility. This in turn affects the healing ability of the anastomosis, and neoadjuvant radiotherapy is a high-risk factor for postoperative anastomotic leak in colorectal cancer [44]. For patients at risk of multiple primary colorectal cancers, the preservation of the LCA during the first operation can reserve vascular reserve for later surgery. Patients with PDM mostly have abnormalities of the vascular arch, including the LCA, branches of the SA, and SA in a radial pattern, forming a bear claw-like structure. In addition, the LCA in this group of patients is often short and even, directly forming part of the marginal vascular arch [45, 46]; therefore, not preserving the LCA may lead to extensive intestinal ischemia. In contrast, patients with a high risk of IMA root lymph node metastasis [47] and patients with surgical findings of high anastomotic tension [48] are not recommended for LCA preservation.

Another important factor is the anatomical variation of arteries. Because of a large variation in the anatomical structure of the LCA, a definite theoretical foundation and operational skills are required for surgeons to accurately locate the root of the LCA under laparoscopic guidance and to avoid accidental bleeding when dealing with the vessels. Preoperative mastery of IMA subtyping; accurate determination of the relationship between the LCA, SA, and SRA; and measurement of the distance between the root of the IMA and the beginning of the LCA are essential for successful implementation of LCA-preserving surgery. Therefore, in addition to the routine examination, preoperative patients with colorectal cancer need accurately determined IMA staging by CT angiography and different methods of LCA preservation adopted intra-operatively according to their staging. In this study, we summarized the clinical classifications. Based on the Yada classification and the perspective of the presence or absence of the Riolan artery arch, we conclude that type III is an independent risk factor for the occurrence of anastomotic leakage, and that it is easier to detect free type III cases, which tend to preserve the LCA. For patients without a Riolan artery arch, radical colorectal cancer surgery with preservation of the LCA should be performed. For patients with type III and Riolan artery agenesis, radical colorectal cancer resection with preservation of the LCA should be highly recommended as it offers more advantages for patients’ survival post-surgery. For patients with types I and II, low ligation may be performed for anatomic convenience, but the choice should be made in the context of the patient’s own condition and the intra-operative situation. To better define the surgical plan for the operator pre-surgery, we believe that “IMA-accurate staging” should be incorporated into the standard of care for colorectal cancer surgery, which still requires evidence-based studies with large multicenter samples.

## Conclusion

After fully assessing factors such as patient condition and tumor characteristics, anatomical variation of the IMA and its branches is of great significance for the preservation of the LCA during radical surgery for colorectal cancer. In summary, type III IMA is an independent risk factor for the development of anastomotic leakage and is easier to detect and separate, favoring the preservation of the LCA. In patients with Riolan artery arch agenesis, radical colorectal cancer resection with preservation of the LCA should be performed. For patients with type III IMA and Riolan artery agenesis, radical colorectal cancer resection with preservation of the LCA should be performed, which provides a survival benefit post-surgery. For patients with type I and II IMA, low ligation can be performed for anatomical convenience; however, this is in the context of the patient’s own choices and the prevailing intra-operative conditions.

## Data Availability

Data sharing is not applicable to this article as no datasets were generated or analyzed during the current study.

## Abbreviations

AA: Abdominal aorta
C: Common artery
ILCA: Left inferior colonic artery
IM: Inferior mesenteric artery
IMA: Inferior mesenteric artery
IMV: Inferior mesenteric vein
JSCCR: Japanese Society for Colorectal Cancer Research
LC: Left colonic artery
LCa: Left colic artery
LCA: Left colic artery
LC acc.a: Left middle colonic artery or the left inferior colonic artery
MLCA: Left middle colonic artery
PDM: Persistent descending mesocolon
RST: Rectosigmoid trunk
S: Sigmoid artery
SA: Sigmoid artery (SA)
SR: Superior rectal artery
SRa: Superior rectal artery
SRA: Superior rectal artery
ST: Sigmoid trunk
S trunk: Sigmoid artery trunk
